# miR-122-5p regulates the tight junction of the blood-testis barrier of mice via occludin: miR-122-5p can regulate the tight junction

**DOI:** 10.1186/s12610-021-00126-8

**Published:** 2021-04-08

**Authors:** Limin Liu, Maoying Zhu, Xiaoli Liu, Lumin Fei, Jianyun Shen, Deyu Chen

**Affiliations:** 1 Center for Reproductive Medicine, Huizhou Central People’s Hospital, Huizhou, 516001 Guangdong China; 2 College of Biological and Food Engineering, Fuyang Normal University, No.100 Qinghe Road, Fuyang, 236037 Anhui China

**Keywords:** miR-122-5p, Sp1, Occludine, Jonction serrée, Souris, Cellule de Sertoli, miR-122-5p, Sp1, Occludin, Tight junction, Mice, Sertoli cell

## Abstract

**Background:**

Occludin protein is the primary assembling protein of TJs and the structural basis for tight junction formation between Sertoli cells in the spermatogenic epithelium. The expression of miR-122-5p and occludin are negatively correlated. In order to investigate the regulation mechanism of miR-122-5p on occludin and TJ, the present study isolated primary Sertoli cells from C57BL/6 mice, identified a transcription factor of miR-122-5p in testicle, studied the modulating loci of miR-122-5p on occludin using a dual-luciferase reporter assay, analyzed the regulate of miR-122-5p on the expression of occludin with real-time RT-PCR and Western blot, and studied the effect of miR-122-5p on the tight junction using a Millicell Electrical Resistance System.

**Results:**

The relative luciferase activity in the pcDNA-Sp1 + pGL3-miR-122-5p promoter group was significantly higher than that in the pcDNA-Sp1 + pGL3-basic group, which suggests that transcript factor Sp1 promotes the transcription of miR-122-5p. The relative luciferase activity in the occludin 3′-UTR (wt) + miR-122-5p mimic group was significantly lower than that in the other groups (*p* < 0.01), which indicates that miR-122-5p modulates the expression of occludin via the ACACTCCA sequence of the occludin-3’UTR. The levels of occludin mRNA and protein in the miR-122-5p mimic group were significantly lower than that in the other groups (*p* < 0.05), which indicates that miR-122-5p reduces the expression of occludin. The trans-epithelial resistance of the miR-122-5p mimic group was significantly lower than that of the blank control group after day 4 (*p* < 0.05), which indicates that miR-122-5p inhibited the assembly of the inter-Sertoli TJ permeability barrier in vitro.

**Conclusion:**

These results displayed that miR-122-5p could regulate tight junctions via the Sp1-miR-122-5p-occludin-TJ axis.

## Abstrait

**Contexte:**

La protéine occludine est. la principale protéine d’assemblage des jonctions serrées (JS), et la base structurelle pour la formation de ces jonctions entre les cellules de Sertoli dans l’épithélium séminifère. L’expression de miR-122-5p et de l’occludine sont négativement corrélées. Afin d’étudier le mécanisme de régulation de l’occludine et des TJ par miR-122-5p, nous avons, dans la présente étude, isolé des cellules primaires de Sertoli de souris C57BL/6, identifié un facteur de transcription de miR-122-5p dans le testicule, étudié les loci de miR-122-5p modulants l’occludine par le biais d’un système rapporteur à 2 luciférases, analysé la régulation de miR-122-5p sur l’expression de l’occludine par qRT-PCR et Western blot, et étudié l’effet de miR-122-5p sur les jonctions serrées à l’aide d’un Système de Résistance Electrique Millicell.

**Résultats:**

L’activité relative de la luciférase dans le groupe du promoteur de pcDNA46 Sp1 + pGL3-miR-122-5p était significativement plus élevée que celle observée dans le groupe pcDNA-Sp1 + pGL3-basique, ce qui suggère que le facteur de transcription Sp1 favorise la transcription de miR-122-5p.

L’activité relative de la luciférase dans le groupe 3′-UTR (wt) + miR-122-5p mimant l’occludine était significativement inférieure à celle des autres groupes (*p* < 0,01), ce qui indique que miR-122-5p module l’expression de l’occludine via la séquence ACACTCCA en 3′ UTR de l’occludine.

Les niveaux d’ARNm et de protéine occludine dans le groupe mimant miR-122-5p étaient significativement inférieurs à ceux des autres groupes (*p* < 0,05), ce qui indique que miR-122-5p inhibe l’expression de l’occludine.

La résistance transépithéliale du groupe mimant miR-122-5p était significativement inférieure à celle du groupe témoin vierge après le jour 4 (*p* < 0.05), ce qui indique que miR-122-5p inhibe in vitro l’assemblage des jonctions serrées de la barrière de perméabilité inter-Sertolienne.

**Conclusions:**

Ces résultats montrent que miR-122-5p pourrait réguler les jonctions serrées via l’axe Sp1-miR-122-5p-occludine.

## Introduction

MicroRNAs (miRs) are a family of small noncoding ribonucleic acid (RNA) with a length of ~ 22 bp and carry a 5′-phosphate terminal and 3′-hydroxy terminal. The major functions of miRs include the modulation of transcription, RNA cutting and trimming, messenger ribonucleic acid (mRNA) stabilization and translation, protein stabilization and transportation, chromosome formation and structure stabilization, and cellular development [1]. The role of miRs in spermatogenesis became a hotspot in research of the male reproductive system. Different miRs are involved in the proliferation [2–4] and differentiation of spermatogenic cells [5] and spermatogenesis [6]. Although miRs were closely correlated with the modulation of spermatogenesis, the detailed mechanisms are not clear [7, 8].

miR-122-5p is a transcript processed from the *Hcr* gene, and it plays an important role in modulation of the cell cycle, cell proliferation and apoptosis [9]. miR-122-5p is related to multiple diseases, e.g., it is a prognosis marker for acute heart stroke [10], affects the proliferation of keratinocytes [11], inhibits the migration of melanoma cells [12], suppresses cellular differentiation in nasopharyngeal carcinoma [13], regulates cell proliferation in gastric cancer [14] and correlates with hepatic cancer [9, 15]. However, there are no related reports of miR-122-5p in spermatogenesis.

The blood-testis barrier (BTB) between the seminiferous tubule and blood resides at the level of the myoid layer surrounding the seminiferous tubules, and primarily between Sertoli cells, where tight junctions (TJs) occlude the paracellular spaces at the basolateral membranes between adjacent Sertoli cells [16–18]. TJs are the major structure of the BTB, and abnormal TJs prevent the migration of spermatogenic cells in the seminiferous tubule, which results in a reduction in sperm number [19–21]. Occludin protein is the primary assembling protein of TJs [22] and the structural basis for TJ formation between Sertoli cells in the spermatogenic epithelium. The abnormal opening/resealing of occludin affects spermatogenesis [23]. Our previous study demonstrated a negative correlation between miR-122-5p expression and occludin protein in semen [24]. To further investigate the mechanism of miR-122-5p in the modulation of occludin and TJs, the present study isolated primary Sertoli cells (SCs), identified a transcription factor of miR-122-5p and investigated the modulating locus of occludin by miR-122-5p in SCs of mice, analyzed the effect of miR-122-5p on occludin expression and examined the effect of miR-122-5p on TJs using an in vitro model.

## Materials and methods

### Cell culture and identify

Fourteen-day-old C57BL/6 mice were provided by the Chongqing Enswell Biotechnology Co., Ltd., Chongqing, China. Animals were kept in a room with a 12-h light:1- h dark (12 h–12 h) cycle and temperature of 23–25 °C. Animals were provided food and water ad libitum. The Experimental Animal Ethics Committee of Fuyang Normal University, China approved the experiments (Grant No. 20200006). SCs were isolated from the testicles of 14-day-old C57BL/6 mice, as previously reported [25]. Briefly, the testicle was digested with 0.125% trypsin for 20 min, followed by 0.1% collagenase for approximately 30 min after removal of the capsule. The cell suspension was cultured at 35 °C in a 5% CO_2_ incubator in Dulbecco’s modified Eagle medium/Ham F-12 (DMEM/F12) containing 10% fetal calf serum and 100 U/ml penicillin. After 48 h, the cells were subjected to hypotonic treatment with 20 mM Tris (pH 7.4) to lyse the residual spermatogonia and obtain SCs.

For identify analysis the SCs were fixed with 4% paraformaldehyde solution for immunohistochemical staining. Antigen retrieval was performed at 95 °C in citrate buffer pH 6.0, 6.4 M sodium citrate dihydrate, 1.6 M citric acid monohydrate for 40 min. The slides were cooled at room temperature for 20 min and washed 3 × 3 min with Tris buffer pH 7.6, 0.15 M sodium chloride, 0.05 M Trizma. The slides were block with peroxidase for 5 min and washed as above. The slides were incubated for 30 min with the primary anti-Wilms tumor protein1(anti-WT1) [26] (1:100 dilution, abcam, USA). After washed three times with phosphate buffer saline (PBS) for 3 min each, the horseradish peroxidase-labeled secondary antibody (1:800 dilution, abcam, USA) was added dropwise, then incubated at 37 °C for 30 min, followed by the substrate-chromogen solution (3,3′-diaminobenzidine), and finally counter stained with hematoxylin.

### Analysis of miR-122-5p transcript factor in testicle

Transcription factors that bound the miR-122-5p promoter were predicted using the JASPAR database [27] with the 3 requirements: 1) binding in the promoter core, 2) higher score and 3) correlation with the BTB.

One day prior to transfection, the SCs of mice were inoculated in 24-well plates at a density of 1 × 10^5^ cells/well after digestion with trypsin and cultured with DMEM in 5% CO_2_ incubator at 35 °C. After removal of the medium, the transfection reagent POLO3000 (Shanghai R&S Biotechnology Co. Ltd., Shanghai, China) was added (3.0 μL/well) together with 25.0 μL of culture medium. A mixture of plasmid (Table 1) or culture medium (21.0 μL) was added according to different treatments: Group A (blank control) with culture medium; Group B with plasmids of pcDNA3.1 + pGL3-basic+pRL-TK (pcDNA3.1 basic group); Group C with plasmids of pcDNA3.1+ pGL3-miR122-5p promoter + pRL-TK (pcDNA3.1 miR-122-5p promoter group); Group D with plasmids of pcDNA-Sp1 + pGL3-basic+pRL-TK (Sp1 basic group); Group E with plasmids of pcDNA-Sp1 + pGL3-miR122 promoter +pRL-TK (Sp1 miR122-5p promoter group); Group F with plasmids of pcDNA-GATA4 + pGL3-basic+pRL-TK (GATA4 basic group); and Group G with plasmids of pcDNA-GATA4 + pGL3-miR122 promoter +pRL-TK (GATA4 miR122-5p promoter group). The pcDNA3.1 plasmid (20.0 nM) and pGL3-basic plasmid (20.0 nM) were added in 10.0 μL each, and the pRL-TK plasmid (20.0 nM) was added in 1.0 μL. The cells were placed at room temperature for 5 min then in an incubator at 37 °C for 15 min. After transfection for 6 h, the culture medium was discarded and replaced with fresh medium, and the cells were continuously cultured in the incubator. After 48 h of transfection, the transfection ratio was examined under fluorescence microscopy. The cells were rinsed with PBS 2–3 times and incubated with a lysis solution (100 μL/well) on ice for 15–20 min. The activity of firefly luciferase and Renilla luciferase was examined using a dual-luciferase reporter assay. Each experiment was repeated three times using different batches of SCs.

**Table 1:** Resources of plasmids used in the study

Plasmid name	Resource	Characteristics
pGL3-miR-122-5p promoter	Shanghai R&S Biotechnology Co. Ltd. (Shanghai, China)	Sequence of miR-122-5p promoter was artificially synthesized and cloned into pGL3 with primer: F,ACTTAACGCGTCCGTGGTCCAGGTGAGTGTC;R: GCCTAAGCTTCTGCTAAGGAAAGTCTGTCAGGC.
pcDNA3.1-Sp1	Shanghai R&S Biotechnology Co. Ltd. (Shanghai, China).	CDS of *Mus* Sp1(Gene ID: 20683) was artificially synthesized and cloned into pcDNA3.1 with primer: F:AAGCTTGCCACCATGAGCGACCA, R:GATATCTTAGAAACCATTGCCACTGATATTAATGGA.
pcDNA3.1-GATA4	Shanghai R&S Biotechnology Co. Ltd. (Shanghai, China).	CDS of *Mus* GATA4 (Gene ID: 14463) was artificially synthesized and cloned into pcDNA3.1with primer: F, GGATCCGCCACCATGTACC, R: TCTAGATTACGCGGTGATTATGTCC.
Occludin-3’UTR (wt)	Artificially synthesized by Shanghai R&S Biotechnology Co. Ltd. (Shanghai)	Mouse Ocln ENSMUST00000069756, 3’UTR, length: from 1 to 1500
Occludin-3’UTR(mu)	Artificially synthesized by Shanghai R&S Biotechnology Co. Ltd. (Shanghai)	The mutant occludin-3’UTR lost a fragment of ACACTCCA at 210–217 (Mouse Ocln ENSMUST00000069756, 3’UTR, length: from 1 to 1500).
pcDNA3.1	Promega (Madison, USA)	
pRL-TK	Promega (Madison, USA)	
pGL3-basic	Promega (Madison, USA)	
miR-122-5p mimic/inhibitor siRNA	Guangzhou RiboBio Co., Ltd. (Guangzhou, China).	
miR-122-5p mimic/inhibitor siRNA NC	Guangzhou RiboBio Co., Ltd. (Guangzhou, China).	

1. UTR:Untranslated Region

2. siRNA: Small interfering RNA

### Effect of miR-122-5p on the expression of occludin

The SCs of mice were cultured in 5% CO_2_ at 37 °C. One day prior to transfection, the cells were digested with trypsin and inoculated in 24-well plates (1 × 10^5^ cells/well). For transfection, the medium was replaced by a mixture (3.0 μL of transfection reagent, 0.5 μL of 100.0 nM miR-122-5p and 50.0 μL medium) with the following plasmids (0.5 μL at 100.0 nM; Table 1): Blank control group with only medium; miR-122-5p mimic negative control (NC) group; miR-122-5p mimic group; miR-122-5p inhibitor NC group; and miR-122-5p inhibitor group. The cells were cultured in an incubator after gentle shaking, and the medium was replaced with fresh medium after 6–8 h of transfection. The cells were collected after 48 h of transfection. Total RNA was isolated using TRIzol (Invitrogen, Carlsbad, CA) according to manufacturer’s instructions. Total RNA concentration was quantified by absorbance at 260 nm using a SmartSpec 3000 spectrophotometer (Bio-Rad, Hercules, CA). RNA integrity was assessed using the Agilent 2100 Bioanalyzer (Agilent Technologies, Santa Clara, CA) and samples with RNA integrity number (RIN) > 9 were used in the studies.

The qRT-PCR was performed using SYBR® Green I and the TransStart Green qPCR SuperMix in a Lightcycler 96 (Roche, US) according to the manufacturer’s instructions. Briefly, 500 ng of total RNA obtained from SCs was reverse-transcribed using Megaplex reverse transcription (RT) Primers and the TaqMan miRNA reverse transcription kit in a total of 7.5 μL volume. The primers for occludin were forward, AGACCTGATGAATTCAAACCCA and reverse, CCACACAGGCAAATATGGCG. The polymerase chain reaction (PCR) system consisted of 2 x SYBR® Green Mix (10.0 μL, Roche, US), primer mix (1.0 μL), template cDNA (5.0 μL) and ddH_2_O (4.0 μL). The following reaction conditions were used: 2 min of initial denaturation at 95 °C followed by 40 cycles of 15 s at 95 °C for denaturation, 20 s at 60 °C for annealing and 20 s at 72 °C for extension. Expression was calculated using the 2 ^- △△Ct^ method. The primers of miR-122-5p were forward, CCTGAGTGTGACAATG and reverse, GAGCAGGCTGGAGGA. The reaction system consisted of 2x SYBR® Green Mix (10.0 μL, Roche, US), primer mix (1.0 μL), template cDNA (5.0 μL) and ddH_2_O (4.0 μL). The reaction conditions for qRT-PCR included initial denaturation at 94 °C for 4 min followed by 35 cycles of denaturation at 94 °C for 20 s, annealing at 60 °C for 30 s and extension at 72 °C for 30 s. U6 was used as internal control. The expression ratio was calculated using the 2 ^- △△Ct^ method [26].

The total protein was extracted according to the manufacturer’s instructions. Total protein concentrations were measured using the bicinchoninic acid (BCA) method, boiled with 5x loading buffer at 100 °C for 10 min, centrifuged and separated using SDS-PAGE electrophoresis under 100 V for 1.5 h. The proteins were transferred to NC membranes under 300 mA constant current and incubated with 50 mL trimethylaminomethane tween (TTBS) (20.0 mmol/L Tris, pH 7.5, 0. 5 g/L Tween 20, 8.0 g/L NaCl) containing 50 g/L skim milk for 2 h. The NC membrane was incubated with a primary mouse occludin antibody (ab216327, 1:2000, Abcam) or ß-actin antibody (ab179467, 1:2000, Abcam) at 4 °C overnight. Membranes were washed with TTBS 3 times for15 min and incubated with an HRP-conjugated rabbit anti-mouse IgG (111–035-008, 1:2000, Jackson) at room temperature for 2 h. The NC membrane was rinsed with TTBS 3 times, 10 min each time, incubated with novaECL for 1 min and exposed to film in the dark. The band density was scanned using a digital gel imaging system, and the gray value of bands was measured. The expression of occludin was calculated as the ratio of occludin to ß-actin, which served as the internal referral. Each experiment was repeated three times using different batches of Sertoli cells.

### Analysis of potential modulation site of miR-122-5p on occludin-3’UTR

The potential targeting site of miR-122-5p modulation on the occuldin-3’UTR was analyzed online using TargetScanMouse (http://www.targetscan.org/cgi-bin/mmu_71/view_gene.cgi?rs=ENSMUST00000069756.5&taxid=10090&members=miR-122-5p&subset=1&showcnc=0&shownc=0&shownc_nc=0&showncf1=0&showncf2=0). The results indicated one potential miR-122-5p modulation site located in the 210–217 region of the occuldin-3′ UTR. Wild-type and mutated occuldin-3′ UTR (Mouse Ocln ENSMUST00000069756, 3′ UTR, length: from 1 to 1500) were synthesized by Shanghai Sangon. In contrast to the wild-type occuldin-3′ UTR, the mutant occludin-3′ UTR lost ACACTCCA at 210–217.

The SCs of mice cultured in an incubator with 5% CO_2_ at 37 °C were treated with trypsin 1 day prior to transfection and inoculated in a 24-well plate (1 × 10^5^ cells/well). The transfection system added to each well was mixed with 3.0 μL transfection reagent POLO 3000 (Shanghai R&S Biotechnology Co. Ltd., Shanghai, China), 0.5 μL plasmid (Table 1) at 20.0 nM and 50.0 μL culture medium. The cells were treated differently according to the following groups: Group A with vehicle (blank control); Group B with occludin 3′-UTR (Wt, WT); Group C with occludin 3′-UTR (wt) + miR-122-5p mimic NC (Wt + Mimic NC); Group D with occludin 3′-UTR (wt) + miR-122-5p mimic (Wt + Mimic); Group E with occludin 3′-UTR (Mu) (Mu); Group F with occludin 3′-UTR (mu) + miR-122-5p mimic NC (Mu + Mimic NC); and Group G with occludin 3′-UTR (mu) + miR-122-5p mimic (Mu + Mimic). The medium was replaced with fresh medium after 8 h of culture in an incubator at 37 °C. The cells were rinsed with PBS 3 times, and 100.0 μL 1× lysis buffer was added for 15–20 min on ice after 48 h of transfection. After 48 h of transfection, the transfection ratio was examined under fluorescence microscopy. The lysate was collected and measured for the activities of luciferase using the Dual-Luciferase Reporter Assay System (Promega, E1910), according to the manufacturer’s instructions. Each experiment was repeated three times using different batches of Sertoli cells.

### Assessing the integrity of inter-Sertoli TJ permeability barrier by measuring the TER across Sertoli cell epithelium

To examine the effects of miR-122-5p on the assembly and maintenance of inter-Sertoli TJ permeability barriers in vitro, primary Sertoli cells were isolated as described above and cultured at 1.2 × 10^6^ cells/cm^2^ on Matrigel (1:7)-coated bicameral units (Millipore Corp., Bedford, MA), as previously described [28]. TJ assembly was monitored by trans-epithelial resistance (TER) across the Sertoli cell epithelia using a Millicell Electrical Resistance System, as previously described [29]. The SCs of mice were cultured with DMEM/F12 containing 10% fetal calf serum and 100 U/ml penicillin in an incubator with 5% CO_2_ at 37 °C. One day prior to transfection, the cells were digested with trypsin and inoculated at a concentration of 1.2 × 10^6^ cells/cm^2^ in apical chambers of the bicameral units with 0.5 ml medium. The same medium (0.5 ml) was added to the basal chambers. After 12 h of culture in an incubator with 5% CO_2_ at 37 °C, the medium was replaced with fresh antibiotic-free medium. After another 6 h of culture, a mixture of 3.0 μL transfection reagent and 50.0 μL culture medium were added to each well, followed by 0.5 μL miR-122-5p mimic (50.0 nM) or miR-122-5p inhibitor (Table 1). After 6–8 h of transfection, the medium was replaced with fresh medium every 2–3 days. The TER was measured on days 1, 2, 3, 4, 5, 6, 7 and 8. Each experiment was repeated three times using different batches of Sertoli cells.

### Statistical analysis

All data are expressed as the means ± SD. Statistical analysis was performed using GraphPad Prism 6. All experimental data were normally distributed. Differences between two groups were examined using *t* - test. Differences among different groups were examined using one-way ANOVA. The significant difference level was set as *p* < 0.05.

## Results

### Identification of SCs

**Fig. 1:**
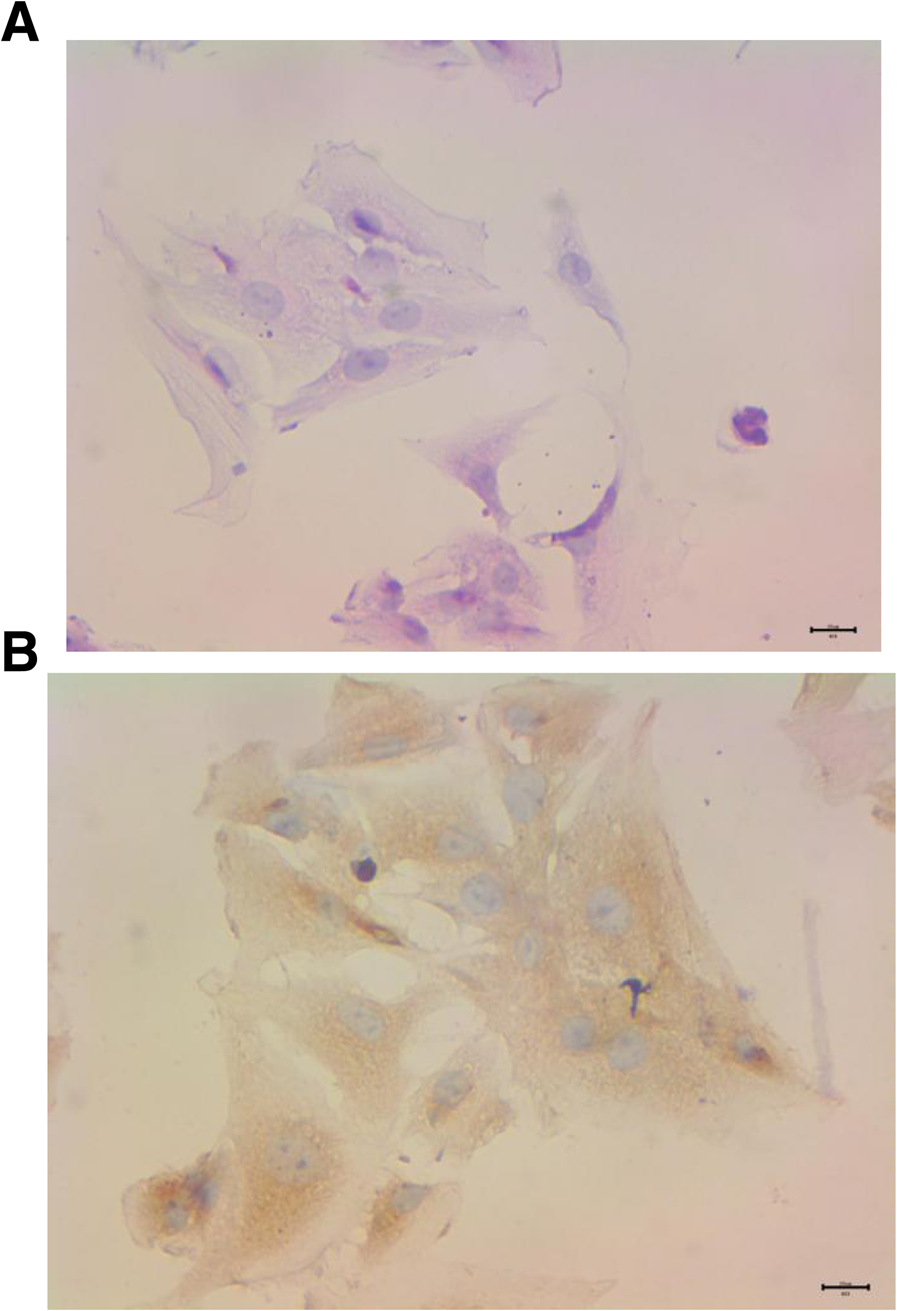
For Morphological analysis, the SCs were stained with hematoxylin-eosin. The results were showed in (**a**). The cell morphology was irregular. The nucleus was blue and the cytoplasm was pink. For identify analysis the SCs were performed with immunohistochemical staining. The results were showed in (**b**). The cells were filled with WT1 which was one of marker protein of SCs. The results suggested that the isolated cells were SCs. Bar = 0.2 μm. SC:Sertoli cell

The number of SCs in the testis determines both testis size and daily sperm production. Only immature SCs proliferate, so the final number of SCs was determined before adulthood. Sertoli cells proliferation occurs between days 12–15 in rodents [30]. So fourteen -day-old mice were selected in this study. For morphological analysis, the SCs were stained with hematoxylin-eosin. The results are showed in Fig. 1(a). The cell morphology was irregular. The nucleus was blue and the cytoplasm was pink. For identify analysis, the SCs were performed with immunohistochemical staining. The results were showed in Fig. 1(b). The cells were filled with WT1 which was one of marker protein of SCs [26]. The results suggested that the isolated cells were SCs.

### Selection of transcript factor of miR-122-5p

First, JASPAR was used to predict the transcription factors that could bind to the mmu-mir-122-5p promoter. The results showed that 220 transcription factors could bind to the mmu-mir-122-5p promoter. Then, NCBI database was used to find which of the 220 transcription factors were related to BTB. The results showed that 14 transcription factors were closely related to BTB. Finally, Promoterscan was used to predict the core region of the promoter and to analyse which of the 14 transcription factors were able to bind near the core region of the promoter (1 K ~ 1.3 K) with a score greater than 9. After the above analysis, Sp1 and GATA4 were selected as the transcription factors of mmu-mir-122-5p in this study.

### Identification of transcript factor of miR-122-5p in SCs

**Fig. 2:**
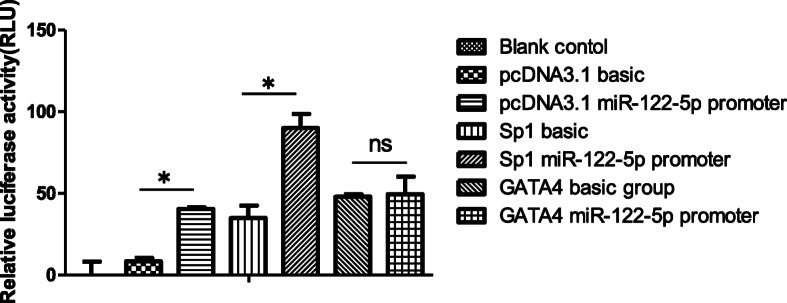
Identification of transcription factors of miR-122-5p in SCs. The fluorescence test indicated that the relative luciferase activity of the Sp1 miR-122-5p promoter group was significantly higher than the Sp1 basic group, which suggests that transcription factor Sp1 promoted the transcription of miR-122-5p (*p* < 0.05). There was no difference between the GATA4 miR-122-5p promoter group and GATA4 basic group (*p* > 0.05), which suggests that GATA4 did not promote the transcription of miR-122-5p. Differences between two groups were examined using *t* - test. *p < 0.05, SEM (*n* = 3), ns p > 0.05. RLU: relative light units

To verify the miR-122-5p promoter, pcDNA3.1 + pGL3-basic or pcDNA3.1 + pGL3-miR-122 promoter were cloned into SCs, and the relative luciferase activity was detected using the dual-luciferase reporter assay. The difference between two groups was compared with *t*-test. The relative luciferase activity in the pcDNA3.1 + pGL3-miR-122-5p promoter group and pcDNA3.1 + pGL3-basic group was 40.5063 ± 1.0126 and 8.6076 ± 1.8481. The value in the pcDNA3.1 + pGL3-miR-122-5p promoter group was significantly higher than the pcDNA3.1 + pGL3-basic group (*p*<0.05). This result suggested that the luciferase reporter plasmid of miR-122-5p promoter was successfully established. To analyze the transcriptional activity of Sp1 and GATA4 on miR-122-5p, Sp1 and GATA4 were colonized into pcDNA3.1 to construct pcDNA-Sp1 and pcDNA-GATA4 plasmids, respectively, then the recombined plasmids of pcDNA-Sp1 + pGL3-basic and pcDNA-Sp1 + pGL3-miR-122 promoter were cloned into SCs. The fluorescence test indicated that the relative luciferase activity in the pcDNA-Sp1 + pGL3-miR-122 promoter group was significantly higher than the pcDNA-Sp1 + pGL3-basic group. However, the difference between the pcDNA-GATA4 + pGL3-basic plasmid group and pcDNA-GATA4 + pGL3-miR-122-5p promoter plasmid group was not significant (Fig. 2). These results suggested that SP1, but not GATA4, was a transcription factor of miR-122-5p.

### Potential modulating locus of occludin-3’UTR by miR-122-5p

**Fig. 3:**
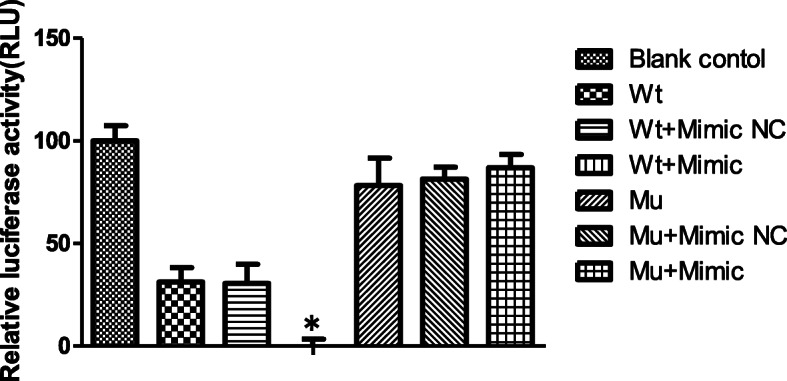
Modulation locus of the occludin-3’UTR by miR-122-5p. The synthesized wild-type and mutant occludin-3’UTR with miR-122-5p were cloned into SCs. The results indicated that the relative luciferase activity in the occludin 3′-UTR(wt) + miR-122-5p mimic group was significantly lower than the other groups (*p* < 0.01). Mutation of the miR-122-5p target site partially rescued luciferase activities. The differences between the occludin 3′-UTR(mu) + miR-122-5p mimic group, occludin 3′-UTR(mu) + miR-122-5p mimic NC group and occludin 3′-UTR(mu) group were not significant (*p* > 0.05). These results indicated that miR-122-5p modulated the expression of occludin via the ACACTCCA sequence in the occludin-3’UTR. Differences between two groups were examined using *t* - test. *p < 0.05, Error bars, SEM (*n* = 3). NC: negative control, SC:Sertoli cell, UTR: Untranslated Region; RLU: relative light units

To investigate whether the CACTCCA sequence of the occluding-3’UTR was the modulating locus for miR-122-5p, the synthesized wild-type or mutated occludin-3’UTR with miR-122-5p were cloned into SCs. The results indicated that occludin-3’UTR (wt) or occludin-3ÚTR(mu) cotransfection with miR-122-5p into SCs increased fluorescence expression during continuous observation under an inverted fluorescence microscope in a time-dependent manner and reached a plateau at 48 h, with a transfection rate of 90%. As shown in Fig. 3, dual-luciferase measurement indicated that the relative luciferase activity in the occludin 3′-UTR(wt) + miR-122-5p mimic group was significantly lower than the other groups (*p* < 0.01). Mutation of the miR-122-5p target site partially rescued luciferase activities. The differences between the occludin 3′-UTR(mu) + miR-122-5p mimic group, occludin 3′-UTR(mu) + miR-122-5p mimic NC group and occludin 3′-UTR(mu) group were not significant (*p* > 0.05). These results indicated that miR-122-5p modulated the expression of occludin via the ACACTCCA sequence in the occludin-3’UTR.

### The effect of miR-122-5p interference on occludin expression

**Fig. 4:**
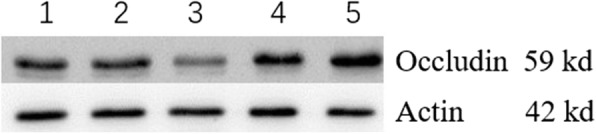
The expression of occludin in SCs. 1. Blank control group; 2. miR-122-5p mimic NC group; 3. miR-122-5p mimic group; 4. miR-122-5p inhibitor NC group; 5. miR-122-5p inhibitor group. The occludin protein in the miR-122-5p mimic group was significantly lower than the other groups (*p* < 0.05). The occludin protein in the miR-122 inhibitor group was significantly higher than the other groups (*p* < 0.05). These results indicated that miR-122-5p reduced the expression of occludin. SCs: Sertoli cells

To investigate the interfering effect of miR-122-5p on the expression of occludin in SCs, the miR-122-5p mimic NC, miR-122-5p mimic, miR-122-5p inhibitor NC and miR-122-5p inhibitor were transfected into SCs for 48 h, and the total proteins and total RNAs were extracted. The expression of miR-122-5p and occludin mRNA in SCs was measured using qRT-PCR, and occludin protein was measured using Western blots. The results indicated that the expression of miR-122-5p in the miR-122-5p mimic group was 1.5-fold higher than the blank control group, 2.43-fold higher than the miR-122-5p inhibitor group, and significantly higher than the other groups (*p* < 0.01) (Table 2). Occludin mRNA and protein in the miR-122-5p mimic group were significantly lower than the other groups (*p* < 0.05). The expression of occludin mRNA and occludin protein in the miR-122 inhibitor group was significantly higher than the other groups (*p* < 0.05) (Fig. 4). These results indicated that miR-122-5p reduced the expression of occludin in SCs.

**Table 2:** The expression of miR-122-5p and occludin in SCs

	miR-122-5p(%)	*Occludin*(mRNA,%)	Occludin(Protein,%)
Blank control	100	100	100
miR-122-5p mimics NC	97.66 ± 3.568	76.209 ± 12.694	99.879 ± 1.195
miR-122-5p mimics	147.37 ± 10.31†	62.844 ± 1.268†	63.448 ± =2.394†
miR-122-5p inhibitors NC	105.54 ± 5.567	69.356 ± 13.568	111.346 ± 34.123
miR-122-5p inhibitors	60.52 ± 4.691†	208.715 ± 11.354†	148.921 ± 12.616†

The expression value of the blank control group was set as 100%. The value of each other group was represented as fold changes (% of control). The difference between two groups was compared with *t*-test. †: *p* < 0.05

*mRNA* messenger Ribonucleic Acid

*NC* negative control

*SCs* Sertoli cells

### Expression of mir-122-5p by Sertoli cells correlates with the assembly of the inter-Sertoli TJ permeability barrier in vitro

**Fig. 5:**
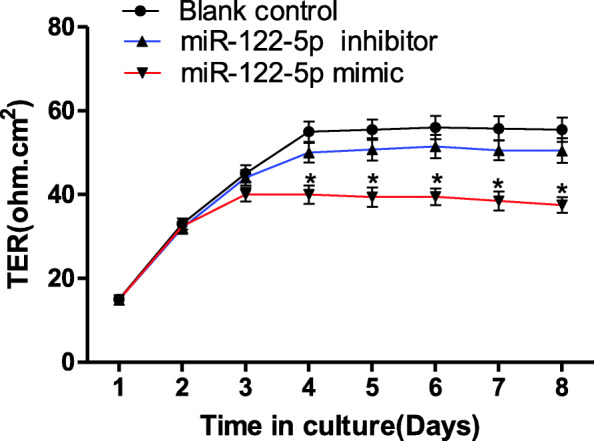
The effect of miR-122-5p on the inter-Sertoli TJ permeability barrier in vitro. The miR-122-5p mimic and inhibitor were transfected into SCs. The functionality of SCs on the TJ barrier was quantified as the TER across the Sertoli cell epithelium on Matrigel-coated bicameral units, and the TER was measured on days 1, 2, 3, 4, 5, 6, 7 and 8. There were no differences between the three groups from day 1 to day 3 (*p* > 0.05). No difference was found between the blank group and miR-122-5p inhibitor group from day 4 to day 8 (*p* > 0.05). However, the TER of the miR-122-5p mimic group was significantly lower than the other two groups beginning on day 4 (*p* < 0.05). This result suggests that the miR-122-5p mimic inhibits the assembly of the inter-Sertoli TJ permeability barrier in vitro. The difference between two groups was compared with *t*-test. *p < 0.05, Error bars, SEM (*n* = 3). TER: trans-epithelial resistance

To investigate whether miR-122-5p modulated the TJs, the miR-122-5p mimic and inhibitor were transfected into SCs. The function of SCs on the TJ barrier were quantified using the TER across the SC monolayers on Matrigel-coated bicameral units. The TERs were measured on days 1, 2, 3, 4, 5, 6, 7 and 8. Data are shown in Fig. 5. There were no differences between the three groups from day 1 to day 3 (*p* > 0.05). No difference was found between the blank group and miR-122-5p inhibitor group from day 4 to day 8 (p > 0.05). However, the TER of the miR-122-5p mimic group was significantly lower than the other two groups after day 4 (*p* < 0.05). This result suggested that the miR-122-5p mimic inhibited the assembly of the inter-Sertoli TJ permeability barrier in vitro.

## Discussion

There are numerous reports on miR-122-5p from other laboratories [29–32], and our previous study demonstrated the correlation between the expression of miR-122-5p and occludin protein in sperm and exfoliative cells of semen [24]. To further investigate the modulating mechanism of miR-122-5p on occludin, an miR-122-5p mimic and miR-122-5p inhibitor were transfected into SCs. The results indicated that miR-122-5p mimic significantly decreased the expression of occludin mRNA and protein, which suggests the miR-122-5p modulates the transcription and translation of occludin. Occludin is an important assembling protein of TJs [31], and it is the basis of TJ formation for Sertoli cells of the spermatogenetic epithelium and determines the normal process of spermatogenesis [32]. The present study indicated that miR-122-5p modulated the expression of occludin via the ACACTCCA sequence of the occludin-3’UTR.

TJs are the major component of the BTB [16], and abnormal TJs affect spermatogenesis [19, 33]. Our previous study indicated that the expression of miR-122-5p correlated with spermatogenesis. Therefore, we hypothesized that miR-122-5p would modulate spermatogenesis via modulation of TJ in the BTB. The present study constructed an in vitro cellular model in which TJs caused a differential in permeability (TER; Fig. 5) on day 4 between the experimental groups and the control group [34]. The miR-122-5p mimic or miR-122-5p inhibitor was transfected into the SCs of mice, and the TER at different times was measured. The results showed that the miR-122-5p mimic inhibited assembly of the inter-Sertoli TJ permeability barrier in vitro, which suggests that miR-122-5p modulates the formation of TJs. This modulation may be one mechanism of the modulation of spermatogenesis.

Sp1 belongs to the Sp protein family (Sp1 ~ Sp9), and it is a transcription factor that exhibits the strongest activity. Sp1 modulates the transcription of genes and multiple posttranslational modifications, including phosphorylation, methylation, glycosylation and acetylation [35, 36]. Sp1 is also closely correlated with spermatogenesis via modulation of the expression of multiple genes during cellular proliferation and embryonic development [37]. A transcription factor in the zinc-finger region of Sp1 binds to the GC or GT elements of target gene promoters in many male germ cells, and these promoters are expressed during the spermatogenesis [38]. Regions enriched with glutamine and serine/threonine in Sp1 coordinate with the modulator to produce a polyprotein preinitiation complex, which is very important to the mediation of transcriptional activation in male germ cells [39, 40]. Sp1 is involved in the modulation of the activities of the occludin promoter by Krüpple-like factor 4 in the blood-tumor barrier. The present study indicated that Sp1 enhanced the activity of miR-122-5p. Because Sp1 is related to spermatogenesis and modulates the transcription of miR-122-5p and occludin, we hypothesized that Sp1 would modulate TJs via modulation of the transcriptional activity of miR-122-5p.

## Conclusion

In summary, the present study indicated that Sp1 enhanced the transcriptional activity of miR-122-5p, inhibited the expression of occludin via the ACACTCCA sequence in the occludin-3’UTR and decreased the formation of TJs in the BTB. Therefore, miR-122-5p can regulate spermatogenesis via the Sp1-miR-122-5p-occludin-TJ axis.

## Data Availability

Yes

## Abbreviations

anti-WT1: Anti-Wilms tumor protein1
BCA: Bicinchoninic acid
BTB: Blood-testis barrier
DMEM: Dulbecco’s modified Eagle medium
miRs: MicroRNAs
Mrna: Messenger Ribonucleic Acid
NC: Negative control
PBS: Phosphate buffer saline
TJ: Tight junction
PCR: Polymerase Chain Reaction
RNA: Ribonucleic Acid
RT: Reverse transcription
SC: Sertoli cell
TER: Trans-epithelial resistance
TTBS: Trimethylaminomethane tween
